# Autoimmune lymphoproliferative immunodeficiencies (ALPID) in childhood: breakdown of immune homeostasis and immune dysregulation

**DOI:** 10.1186/s40348-023-00167-1

**Published:** 2023-09-13

**Authors:** Vasil Toskov, Stephan Ehl

**Affiliations:** 1 Centre for Pediatrics and Adolescent Medicine, Medical Center, Faculty of Medicine, University of Freiburg, Freiburg, Germany; 2 Institute for Immunodeficiency, Center for Chronic Immunodeficiency (CCI), Medical Center, Faculty of Medicine, University of Freiburg, Freiburg, Germany

**Keywords:** Autoimmune lymphoproliferative immunodeficiencies, Inborn errors of immunity, Immune dysregulation, Pathogenesis, Targeted therapy

## Abstract

Many inborn errors of immunity (IEI) manifest with hallmarks of both immunodeficiency and immune dysregulation due to uncontrolled immune responses and impaired immune homeostasis. A subgroup of these disorders frequently presents with autoimmunity and lymphoproliferation (ALPID phenotype). After the initial description of the genetic basis of autoimmune lymphoproliferative syndrome (ALPS) more than 20 years ago, progress in genetics has helped to identify many more genetic conditions underlying this ALPID phenotype. Among these, the majority is caused by a group of autosomal-dominant conditions including CTLA-4 haploinsufficiency, STAT3 gain-of-function disease, activated PI3 kinase syndrome, and NF-κB1 haploinsufficiency. Even within a defined genetic condition, ALPID patients may present with staggering clinical heterogeneity, which makes diagnosis and management a challenge. In this review, we discuss the pathophysiology, clinical presentation, approaches to diagnosis, and conventional as well as targeted therapy of the most common ALPID conditions.

## Introduction

Lymphoproliferation and autoimmunity are relevant manifestations of immuno-hematological diseases. While lymphadenopathy or autoimmune cytopenia in isolation are relatively common and, in most cases, due to secondary causes, the combination of these manifestations raises suspicion for a genetic disease, in particular if associated with other warning signs of an inborn error of immunity (IEI) [1]. These warning signs include clinical manifestations such as increased susceptibility to infection, additional autoimmune manifestations, lymphoma, and indicators raising the likelihood of a genetic disease such as a positive family history, consanguinity or syndromal manifestations, and immunological abnormalities upon laboratory screening. Diagnosis of an underlying IEI is important because of its consequences for prognosis and therapy.

Immune responses are highly dynamic and require tight regulation of proliferation and cell death to maintain homeostasis. Impaired immune homeostasis leads to uncontrolled proliferation of immune cells, manifesting as benign or malignant lymphoproliferation in the form of lymphadenopathy, proliferation of mucosa-associated lymphoid tissue, and hepato- or splenomegaly. Uncontrolled, overactive adaptive immune response may also lead to autoimmunity of almost any organ, and favor immune exhaustion or senescence, paradoxically leading to increased infection susceptibility. It is therefore no surprise that many IEI manifest with hallmarks of both immunodeficiency and autoimmune-lymphoproliferative manifestations [2]. Autoimmune lymphoproliferative syndrome (ALPS) caused by mutations in the genes responsible for the extrinsic apoptotic pathway (*FAS*, *FASLG*, *CASP10*) was the first inherited disease presenting with autoimmunity and lymphoproliferation (autoimmune lymphoproliferative immunodeficiency (ALPID) phenotype) that could be linked to a genetic deficiency [3, 4]. However, ALPS could only explain a part of these cases (around 20%). In recent years, progress in genetics has helped to provide a molecular diagnosis to many more ALPID patients. Next to ALPS, several autosomal-dominant conditions have been identified, which explains another around 20% of cases. They include cytotoxic T lymphocyte-associated antigen-4 (CTLA-4) haploinsufficiency [5, 6], signal transducer and activator of transcription 3 (STAT3) gain-of-function (GOF) disease [7], activated phosphoinositide 3-kinase δ syndrome (APDS) [8–11], and nuclear factor kB1 (NF-κB1) haploinsufficiency [12]. Patients with mutations in more than 50 rare autosomal-recessive genes can also present with an ALPID phenotype (around 10% of cases) [13]. Among these, lipopolysaccharide-responsive vesicle trafficking, beach- and anchor-containing (LRBA) deficiency is particularly notable because of its pathophysiological relationship to CTLA4 haploinsufficiency [14]. Despite all progress, however, half of the patients still cannot be assigned a molecular diagnosis.

In this review, we discuss the pathophysiological basis of the more frequent and mostly autosomal-dominant ALPID conditions and their phenotypic spectrum, as well as laboratory and immunological abnormalities, the approach to diagnosis, and targeted therapies.

### Mechanisms of immune tolerance and homeostasis

The development of a functionally competent adaptive immune system involves the generation of a wide repertoire of B (BCR) and T cell receptors (TCR) during the maturation of B and T cells in the bone marrow and the thymus, respectively. A high percentage of TCRs and BCRs generated on maturing cells has been shown to be self-reactive by recognizing self-antigens. In the bone marrow, for instance, up to 75% of the antibodies generated by immature B cells have been reported to be self-reactive [15]. Depending on the lineage, cell clones with high BCR/TCR affinity for self-antigens may undergo clonal deletion via apoptosis, clonal anergy due to a lack of co-stimulation, clonal diversion to regulatory T cells, or secondary gene rearrangement (receptor editing) [16, 17]. A proportion of self-reactive T cells transforms in an interleukin-2 (IL-2)-dependent manner into CD25 + forkhead box protein P3 (FoxP3) + regulatory T (Treg) cells, which in turn are master effectors of peripheral tolerance [18].

To maintain peripheral immunological tolerance and prevent autoimmunity, the processes of anergy and apoptosis play an essential role. The two-step process of B and T cell activation is tightly regulated. Major histocompatibility complex (MHC) molecules on antigen-presenting cells (APCs) are recognized by TCR on naïve T cells (first signal), which in turn may lead to the formation of an immunological synapse favoring engagement of the co-stimulatory or cytokine receptors (second signal) (e.g., in T cells CD28 binds to the CD80/CD86 ligands on APCs) [19]. If this second signal is missing, a state of hyporesponsiveness (anergy) ensues. CTLA-4 expression on T reg cells can downregulate the CD80/CD86 ligands, thus leading to anergy [20, 21]. The CTLA-4-independent functions of Treg in the maintenance of peripheral tolerance include secretion of inhibitory cytokines and metabolic disruption [22]. Deletion of self-reactive or activated lymphocytes via apoptosis is another important mechanism in the maintenance of immune homeostasis and prevention of autoimmunity [23].

### Autoimmune lymphoproliferative syndrome (ALPS)

Lymphocyte apoptosis contributes to peripheral immune homeostasis by regulating the pool size of certain lymphocyte populations in lymph nodes and spleen. Receptor-mediated apoptosis via the Fas signaling pathway is essential to control unique populations of mammalian targets of rapamycin (mTOR)-dependent hyperproliferative T and B cells, which contain autoreactive specificities. Fas (also called CD95, Fas antigen, Apo-1) is a trimeric receptor of the tumor necrosis factor-receptor (TNF-R) family found on a variety of cells including mature lymphocytes. Upon interaction with its ligand, FasL, the Fas receptor recruits the adaptor Fas-associated death domain (FADD) [24–26]. FADD in turn forms the so-called death-induced signaling complex (DISC) together with pro-caspase-8. Following activation, caspase-8 and caspase-10 then initiate apoptosis [24, 25]. Since apoptosis is induced in a controlled manner through the interaction of a death factor and its receptor, FasL/Fas-induced apoptosis is classified as activation-induced cell death (AICD) [27].

**Fig. 1:**
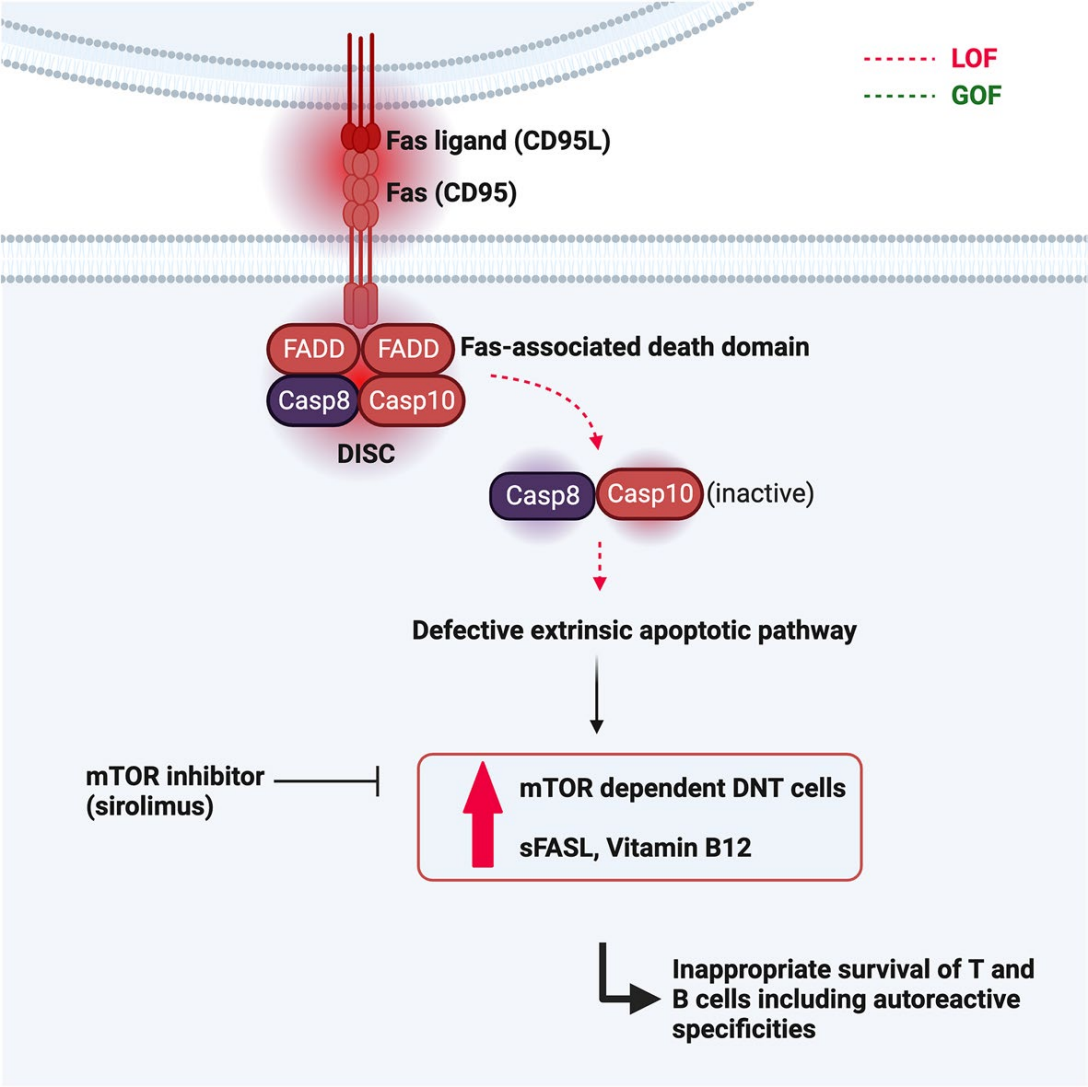
Illustration of the Fas-FasL pathway. Fas (CD95) is a trimeric receptor of the tumor TNF-R family and after binding of the Fas ligand (FasL, CD95L), recruits the adaptor FADD, which in turn forms the so-called DISC together with pro-caspase-8 and pro-caspase-10. Following activation, caspase-8 and caspase-10 then initiate the extrinsic apoptotic pathway leading to proteolysis, DNA degradation, and apoptosis. Mutations in the genes responsible for the Fas-FasL signaling cascade (*FAS*, *FASLG*, *FADD*, *CASP10*) lead to the development of ALPS. Hallmarks of the disease are increased ALPS biomarkers (Vitamin B12 and sFasL), as well as a massive expansion of double-negative T cells (DNT). Increased AKT/mTOR activation leads to DNT hyperproliferation and can be inhibited via mTOR inhibitors such as sirolimus (rapamycin)

Mutations in the genes encoding modules of the Fas-FasL apoptotic pathway (*FAS*, *FASLG*, *FADD*, *CASP10*) lead to ALPS (Fig. 1) [28]. Research into ALPS as a non-malignant, non-infectious cause of uncontrolled lymphocyte proliferation and accompanying autoimmunity was initiated after the first description of dramatic lymphoproliferation in *lpr* strain mice with lupus-like phenotype [29] and the subsequent discovery of deleterious homozygous mutations in the murine FAS receptor [30]. Heterozygous *FAS* mutations affect the intracellular death domain of the protein, but still allow Fas expression on the cell surface, acting dominant-negative because they prevent trimerization and successful DISC formation. They can be either germline (ALPS-FAS) [3, 4] or somatic (ALPS-sFAS). Acquisition of a somatic mutation in hematopoietic progenitor cells leads to growth advantage for Fas-controlled T cells. They accumulate as CD3 + CD4-CD8-TCRab + double negative T cells (DNT) which are enriched for the disease-causing mutation [31, 32]. Isolation of DNA from sorted DNT therefore facilitates diagnosis of somatic variants. Dominant-negative somatic *FAS* mutations underlie around 15% of all ALPS cases [32].

In contrast, heterozygous mutations completely abolishing Fas expression, mostly affecting the extracellular or transmembrane domain, do not act dominant-negative, but cause a 50% reduction of total protein expression [33]. These mutations have a very low penetrance unless combined with an additional, somatic “hit” in the second allele. This might be a somatic missense mutation leading to compound-heterozygous mutations in cells affected by the second “hit,” or loss of heterozygosity (LOH) via uniparental disomy (ALPS-FAS-sLOH) [34, 35]. These second genetic events usually occur in hematopoietic progenitors, but are enriched in DNT to which they confer a selective growth advantage. Rarely, biallelic germline mutations in *FAS* lead to the development of ALPS [33].

Most patients with ALPS due to a *FAS* mutation present at a young age (median age of onset 2, 7 years) with lymphadenopathy, splenomegaly, and autoimmunity, mainly but not limited to autoimmune cytopenia [36]. Patients also frequently show polyclonal hypergammaglobulinemia (IgG and IgA), but can also develop hypogammaglobulinemia. The carrier status of a deleterious *FAS* mutation does not necessarily lead to clinical manifestations, even if such individuals exhibit reduced apoptosis, an increased percentage of DNT, and elevated biomarkers such as sFasL and vitamin B12 [36]. A sometimes massive expansion of DNT is observed in most ALPS patients [4]. Fas-controlled DNT are highly proliferative and secrete high amounts of IL-10, sFASL, and the vitamin B12 carrier protein haptocorin. They can be delineated from conventional DNT by the marker combination CD38 and CD45RA, which are not co-expressed on any other known T cell subset [37]. sFASL and vitamin B12 are excellent diagnostic biomarkers for the disease with positive and negative predictive values above 90% [38–40]. FAS-deficient B cells show an increased propensity to develop into auto-reactive switched memory B cells [41]. The aberrant class-switching explains the hypergammaglobulinemia (IgG and/or IgA) and the reduction in IgM. At the same time, marginal zone B cells are reduced, which leads to an impaired anti-polysaccharide response [42]. In contrast to other ALPID, such as CTLA-4 haploinsufficiency or LRBA deficiency, tissue lymphocytosis in ALPS is mostly confined to the secondary lymphoid organs. On histopathological examination, follicular and paracortical hyperplasia of lymphoid organs with expansion of DNT cells is usually observed [43].

Only a few patients with disease-causing mutations in other components of the Fas pathway have been described in the literature. *FAS ligand* deficiency (ALPS-FASLG) follows an autosomal-recessive inheritance and presents clinically like homozygous ALPS-FAS [44–46]. No convincing disease association of heterozygous *FASLG* mutations has so far been described [47–49]. FADD deficiency also follows an autosomal-recessive mode of inheritance, but the clinical phenotype of this disease is more complex. While patients show variable lymphoproliferation/splenomegaly, they have also been reported to present with recurrent episodes of encephalopathy and invasive pneumococcal disease, as well as severe viral infections [50–52]. This points to additional Fas-independent effects in FADD deficiency. Similarly, *CASP8* mutations cause a more complex combined immunodeficiency. Finally, *CASP10* mutations were initially reported to be associated with ALPS, but the evidence is not convincing so far [53, 54].

Malignancies, mostly B and T cell lymphomas, and rarely non-lymphoid malignancies have been described in patients with ALPS-FAS with median onset in late adolescence or early adulthood [36, 55, 56]. Nonetheless, a long-term survival of ALPS-FAS was estimated to be about 85% by age 50 [36]. Interestingly, non-malignant lymphoproliferation and autoimmune manifestations such as cytopenia may spontaneously improve with age [36, 55].

### CTLA-4 insufficiency and LRBA deficiency

Inhibitory receptors such as CTLA-4 play an important role in immune regulation and peripheral immunological tolerance by inhibiting immune cell activation [21]. CTLA-4 is upregulated on activated T cells and constitutively expressed on FoxP3 + Tregs [57, 58]. Upon T cell stimulation, endosomal CTLA-4 is transported to the cell surface, where it negatively impacts the immunological synapse by outcompeting CD28 for binding to the costimulatory ligands CD80/CD86. Moreover, CTLA4 downregulates costimulation by ripping out CD80/86 from the membrane via a process called trogocytosis or trans-endocytosis [59, 60]. While FoxP3 + Tregs limit autoimmunity, they are also enriched in tumors and impair anti-tumor immunity [61], which can be dramatically improved via immune checkpoint inhibition [62]. CTLA-4 checkpoint inhibitors are currently a part of the standard therapy regimen in many malignancies.

**Fig. 2:**
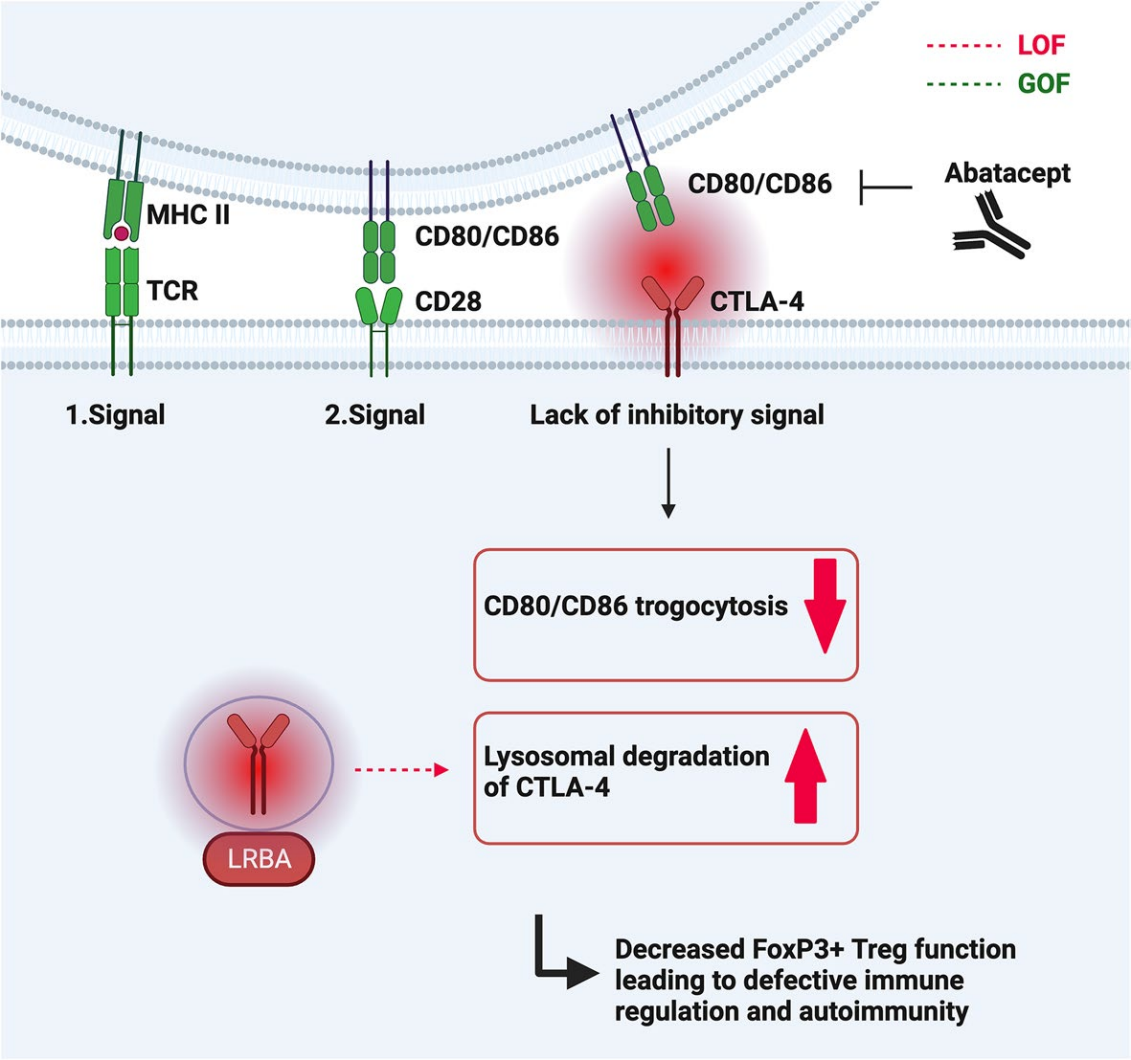
The two-step process of T cell activation. To counteract it, CTLA-4 in endosomes reaches the cell surface, outcompetes CD28, and binds and downregulates CD80/CD86 in a process called trogocytosis. LRBA acts intracellularly to stabilize and protect intracellular CTLA-4 from lysosomal degradation, thus maintaining the pool of available CTLA-4. Both in CTLA-4 haploinsufficiency and LRBA deficiency, lack of CTLA-4 (either due to decreased translation or increased lysosomal degradation) limits the function of regulatory T cells. Abatacept is a soluble CTLA-4 immunoglobulin fusion protein (Fc-region of human IgG1 linked to the extracellular domain of CTLA-4), which mimics CTLA-4 function and can be used successfully as a targeted therapy in both conditions

Heterozygous autosomal-dominant mutations in the *CTLA4* gene in humans were first described in 2014 (Fig. 2). They can be associated with an ALPS-like condition with prominent immune dysregulation and lymphoproliferation [5, 6]. Age of onset can be in early childhood but is often in late adolescence; however, clinical penetrance is variable, since 30–40% of mutation carriers remain broadly asymptomatic. No correlation between specific mutations and the disease severity has been observed [6, 63]. In contrast to ALPS, the lymphocyte accumulation is not limited to secondary lymphoid organs: infiltrates can be seen in diverse organs with predominance of the intestine, brain, and lungs [63]. This is associated with various autoimmune complications including autoimmune cytopenias, enteropathy, thyroiditis, diabetes mellitus type 1, or autoimmune skin conditions [5, 6, 63]. This broad phenotype is explained by the important immune tolerance mechanisms mediated via CTLA-4. Patients with CTLA-4 haploinsufficiency patients frequently fulfill diagnostic criteria for common variable immunodeficiency (CVID) due to a marked decrease in switched memory B cells and hypogammaglobulinemia (IgA, IgG, and/or IgM). This is associated with increased infection susceptibility [63]. Interestingly, a subset of exhausted B cells including self-reactive specificities, called CD21low B cells is frequently also elevated in mutation carriers [5, 6, 63, 64], which highlights the importance of CTLA-4-mediated regulation of humoral immunity and prevention of autoantibody formation [65]. The percentage of FoxP3 + Treg cells is increased, even in asymptomatic mutation carriers [63].

Functional CTLA-4 protein deficiency leading to impaired Treg function, but without mutations in the *CTLA-4* gene, can be observed in LRBA deficiency [66]. The LRBA protein protects intracellular CTLA-4 from lysosomal degradation [66], maintaining the pool of available CTLA-4 protein prior to immune cell activation, thus acting indirectly in concert with CTLA-4 as an immune checkpoint. Hence, biallelic mutations in *LRBA* are associated with a phenotypically similar syndrome of immune dysregulation, lymphoproliferation, hypogammaglobulinemia, enteropathy, and increased infection susceptibility [14, 66]. The disease is more severe with onset in early childhood (median 2 years) and almost complete penetrance. Autoimmune manifestations are more common when compared to CTLA-4 haploinsufficiency [67–70]. The increased penetrance of LRBA deficiency might be due to the even lower total levels of the CTLA-4 protein than in CTLA-4 haploinsufficiency due to its increased lysosomal degradation [69]. In terms of immune phenotype, FoxP3 + Tregs, switched memory B cells and plasmablasts are usually reduced, while CD21low B cells are increased [67, 71].

There are no reliable biomarkers for the diagnosis of CTLA4 haploinsufficiency or LRBA deficiency, rendering genetic analysis the key diagnostic procedure. If mutations of unknown significance are detected, further diagnostic procedures may include LRBA protein expression via flow cytometry or Western blot, or CTLA-4 expression and CTLA-4-dependent trans-endocytosis of CD80 via Treg cells [68].

In a review of published CTLA-4 haploinsufficiency cases, autoimmunity and hypogammaglobulinemia preceded the development of malignancy, with a cumulative incidence of disease manifestations increasing up to 70% at age 40 [72]. In a cohort of 131 patients, 12.9% developed malignancy with a median onset between 32 and 34 years of age, most commonly lymphoma or gastric cancer, with EBV viremia posing a significant risk factor [73]. LRBA deficiency is a more severe disease, although the occurrence of cancer is less frequent [67, 70]. A current report by Tesch et al. showed a 50–60% probability of survival 15–20 years after disease onset, irrespective of the treatment modality [70].

### Germline STAT3 gain-of-function (GOF)

STAT3 is a part of the family of STAT, and as such, a key transcription factor involved in the regulation of multiple immune activation and differentiation pathways [74]. STAT3 is activated by numerous cytokine receptors after binding of their ligand. They include the common gamma chain (IL-2, IL-4, IL-7, IL-9, IL-15, and IL-21), the gp130 (e.g., IL-6, IL-11, IL-27), IL-10 (e.g., IL-10, IL-22), IL-12, IL-23, and interferon (IFNα, IFNβ, and IFNγ) receptor families, as well as receptors for macrophage and granulocyte colony-stimulating factors and hormones such as epidermal growth factor, growth hormone, or insulin-like growth factor. The binding of these cytokines to their specific receptor allows for the activation of an associated Janus Kinase (JAKs) and subsequent phosphorylation of the tyrosine residues on the intracellular domains of the receptor [75]. This in turn leads to the recruitment and phosphorylation of the STAT3 transcription regulator and its homo- or heterodimerization, translocation to the nucleus, and binding to STAT3-responsive DNA sequences which initiates transcription of cytokine-responsive genes and thus a variety of immune responses [75]. Unphosphorylated STAT3 also mediates a variety of non-canonical pathways, such as IL-6-mediated activation of NF-κB [76]. Suppressors of cytokine signaling 3 (SOCS3) and protein inhibitors of activated STAT3 (PIAS3) negatively regulate STAT3 function [77].

**Fig. 3:**
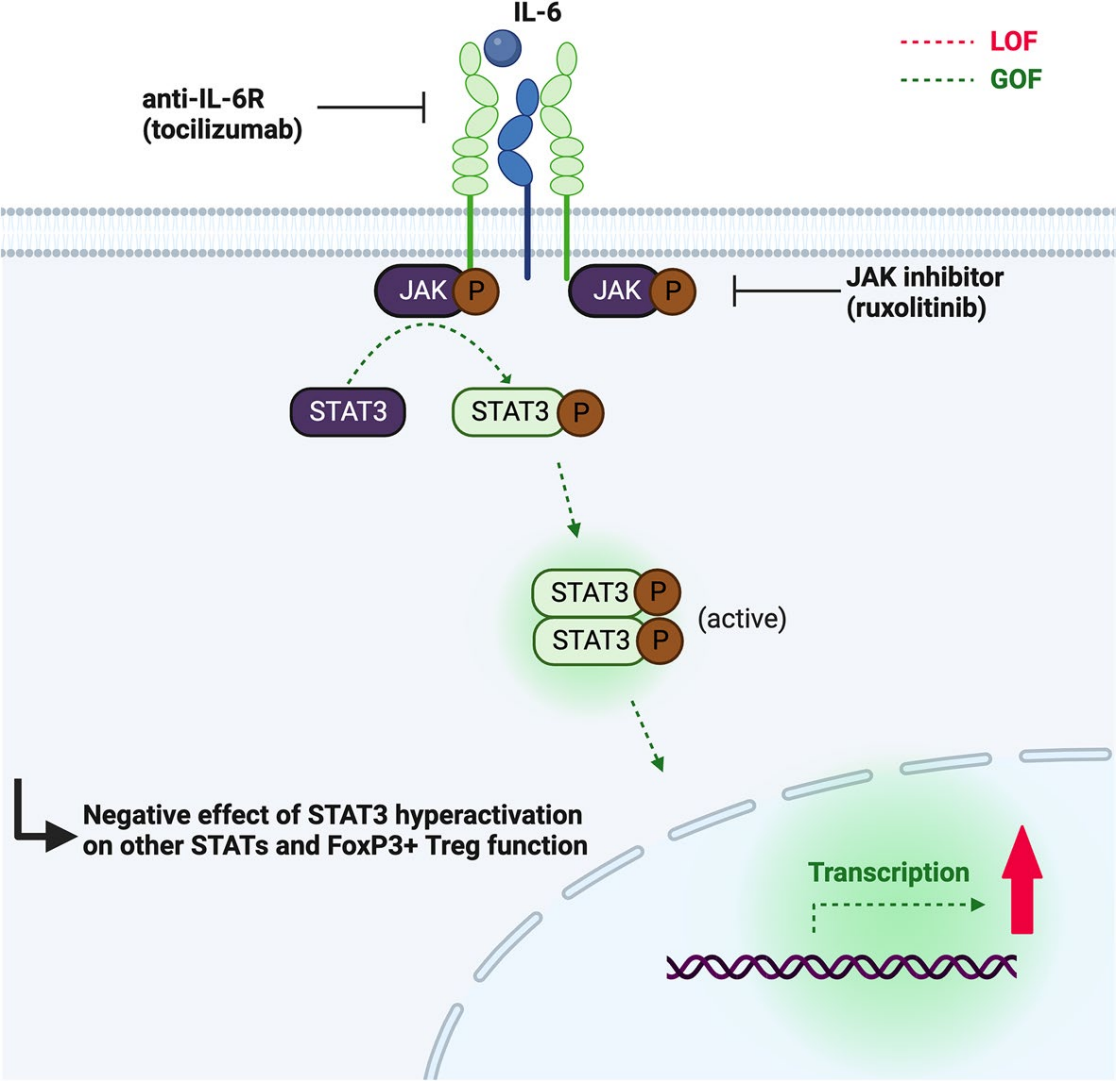
Illustration of the JAK/STAT pathway. After binding to their receptor, cytokines such as IL-6 activate an associated Janus Kinase (JAK), which upon phosphorylation of its tyrosine residues recruits and phosphorylates the STAT3 transcription regulator. Phosphorylated STAT3 in turn forms homo- or heterodimers which translocate into the nucleus and impact the transcription of cytokine-responsive genes. In STAT3 GOF, the signaling pathway can lead to increased phosphorylation, altered dimer formation, as well as changes in gene expression. Targeting molecules which are part of the STAT3 pathway leads to improved STAT3 GOF disease control, e.g., disruption of the IL-6/IL-6R interaction via the anti-IL-6R monoclonal antibody tocilizumab. Another strategy is the inhibition of JAK by jakinibs such as ruxolitinib

The essential function of STAT3 in maintaining immune homeostasis was first shown in hyper-immunoglobulin E syndrome (HIES), a severe immunodeficiency due to dominant-negative mutations in the *STAT3* gene [78, 79]. Somatic activating (GOF) mutations in *STAT3* were first described in relation to malignant lymphoproliferation and often cluster in the SH2 dimerization and activation domain of STAT3 [80, 81]. In contrast, germline STAT3 GOF mutations (Fig. 3) lead to an early-onset disease of poly-autoimmunity and lymphoproliferation and are found in all functional domains of the protein [7, 82–84].

Patients present with variable symptoms, such as lymphoproliferation/splenomegaly, autoimmune cytopenia, type I diabetes in infancy, and enteropathy, as well as short stature and increased susceptibility to viral and bacterial infections [7, 82, 83]. Early severe interstitial lung disease affects some patients, and systemic vasculopathy may also develop [84]. Of note, early-onset diabetes (< 2 years of age) is rarely seen in other IEI from the ALPID spectrum. The age of onset is early (2–3 years of age) with incomplete clinical penetrance and sequential development of disease manifestations [84, 85]. Jägle et al. characterized different *STAT3* GOF mutations and clustered them in three groups depending on their molecular activation mechanisms, which correlated to some extent with the observed variable clinical penetrance [86]. Some of the disease manifestations, such as short stature and susceptibility to infection, can be explained by the effect of constitutive STAT3 activation on the activity of other STATs, e.g., decreased growth hormone-STAT5 and Interferon-STAT1 signaling, respectively [82, 83, 87]. The deficient STAT5-response explains the clinical overlap between *STAT3* GOF and *STAT5B* loss-of-function (LOF)-associated disease [88].

The immune phenotype is not sufficiently characteristic for disease diagnosis. Hypogammaglobulinemia with reduced switched memory and increased CD21low B cells is a common feature, whereas reduced naive CD4 T cells, elevated CD57 + CD8, and DNT cells can be observed in some of STAT3 GOF patients [83, 84, 89]. Interestingly, *STAT3* GOF mutations lead to reduced Treg populations, in line with the known suppressive effect of STAT3 activity on FoxP3 expression and Treg development [90, 91]. Decreased IL-2-mediated STAT5 signaling in STAT3 GOF may also contribute to the observed defect in Tregs [90, 92]. There is no gold standard for functional analyses of *STAT3* mutations, but most frequently, the mutant allele is expressed in a *STAT3*-deficient cell line followed by a luciferase reporter assay to measure its transcriptional activity [86].

In contrast to other disorders of the ALPID spectrum, malignancy seems to occur less frequently in patients with activating germline *STAT3* mutations: in a cohort of 191 patients, 12 developed cancer, most commonly marginal zone B cell and LGL lymphomas [84]. STAT3 GOF patients usually present with very early-onset endocrine and gastrointestinal manifestations, which may be diagnostic clues for the disease, followed by lymphoproliferation, autoimmune cytopenia, and interstitial lung disease [84, 85]. Early diagnosis is essential for survival, since severe cases with enteropathy, autoimmune hepatitis, and oxygen dependence are associated with early death [84]. 

### NF-κB1 haploinsufficiency

NF-κB transcription factors play an essential role as regulators of the innate and adaptive immunity. The NF-κB family consists of NF-κB1 (p50 and its precursor p105), NF-κB2 (p52 and its precursor p100), RelA (p65), c-Rel, and RelB [93–95]. These proteins share a Rel homology domain for DNA binding and dimerization, with a variety of dimers (the two canonical are p50:p65 and p52:RelB) forming in the cytosol [93–95]. At rest, NF-κB dimers are bound to inhibitory IκB proteins in the cytoplasm. Degradation of the IκB proteins occurs through phosphorylation by the IκB kinase (IKK) complex consisting of the catalytically active IKKα and IKKβ, and the regulatory subunit IKKγ (NEMO), and leads to the release of bound NF-κB dimers, which then translocate to the nucleus [93–95]. The canonical NF-κB1 pathway can be activated by a variety of signals from receptors, such as the tumor necrosis factor receptor (TNF-R), antigen, and pattern-recognition receptors (PRR). After IKKβ and NEMO-dependent degradation of IκB, p65-containing heterodimers translocate into the nucleus, where they regulate gene expression [93]. While increased activation of the NF-κB pathways is associated with malignancy [96], loss-of-function mutations affecting NF-κB signaling may lead to a variety of immunodeficiencies [97].

**Fig. 4:**
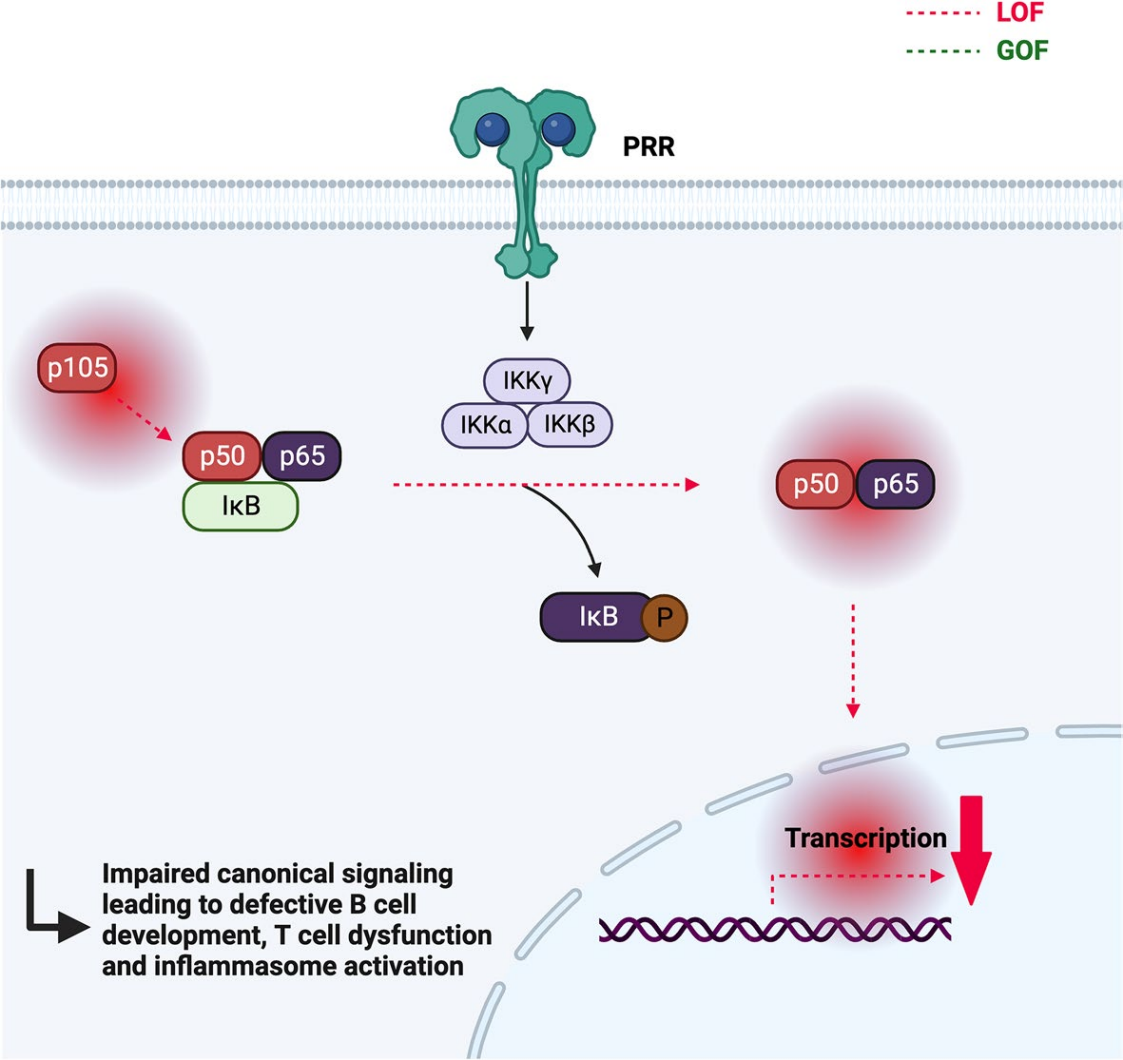
Illustration of the NF-κB pathway. The NF-κB1 transcriptional factor (p50 and its precursor p105) is active upon dimerization (p50:p65) in the cytosol. At rest, NF-κB dimers are bound to inhibitory IκB proteins. After activation (here via PRR signaling), IκB proteins are phosphorylated by the IκB kinase (IKK) complex, which releases the NF-κB dimers. p65-containing heterodimers can then translocate into the nucleus and regulate gene expression. Heterozygous loss-of-function mutations in *NFKB1* are associated with reduced protein levels of the p105 and/or p50 subunit and lead to the development of a complex immunodeficiency

Heterozygous loss-of-function mutations in *NFKB1* associated with reduced protein levels of the p105 and/or p50 subunit cause a complex immunodeficiency (Fig. 4), frequently associated with lymphoproliferation and autoimmune manifestations [12, 98–100]. Age of onset and clinical penetrance are highly variable, with symptoms developing between 6 months and 79 years (median 12 years) of age [100]. In a report by Tuijnenburg et al. about 40% of mutation carriers remained asymptomatic, even though p50 expression was reduced in all carriers [98]. Patients with NF-κB1 haploinsufficiency present most commonly with respiratory infections, hypogammaglobulinemia, autoimmune cytopenia, and organ-specific autoimmunity, as well as lymphoproliferation [12, 98, 100–102]. Necrotizing fasciitis is a rare, but severe and characteristic manifestation of the disease. In some rare cases, episodes of severe autoinflammation with increased production of IL-1 and TNF were observed [99].

In terms of immune phenotype, most mutation carriers showed reduced switched memory B cells [98–100]. Interestingly, increased CD21low B cells may differentiate between symptomatic and asymptomatic carriers [98]. Immunoglobulin class-switching is regulated by NF-κB through various mechanisms [103], e.g., expression of the gene *AICD* (activation-induced cytidine deaminase) [97, 104], which may account for the decreased switched memory B cells and hypogammaglobulinemia (IgM, IgA, and/or IgG) in NF-κB haploinsufficiency. Even though the B cell defect is more pronounced, some patients present with chronic viral infection due to functional impairment of T cell immunity, including reduced effector memory and Th17 cells, as well as impaired proliferative response [99, 101]. Increased activation of the inflammasome and IL-1 secretion led to severe autoinflammation in some patients [99]. Possible pathophysiological mechanisms include reduced activity of the NF-κB-p62-mitophagy regulatory loop [105], decreased p50:p50 homodimers [106], and increased binding between NF-κB1 and IKKγ [99]. Regarding functional analysis of *NFKB1* mutations, there are no simple screening assays. The p105 and/or p50 levels in transfected cells can be measured via Western blot. Moreover, the mutant allele can be expressed in a cell line and transcriptional activity measured via a NF-κB1-responsive reporter assay [100].

*NFKB1* is a tumor suppressor gene, and NF-κB1 haploinsufficiency has been shown to promote tumorigenesis in murine models [107]. Malignancy (T and B cell lymphomas as well as solid tumors) also occurs in human NF-κB1 haploinsufficient patients at a median age of diagnosis of 46 years [100]. Age of onset and disease manifestations including cancer are highly variable even within the same family [12, 98], with clinical penetrance increasing in an age-dependent manner [100]. In a cohort of 121 affected patients, death occurred at a median age of 52 years [100].

### Activated phosphoinositide 3-kinase δ syndrome (APDS)

Class I phosphoinositide 3-kinases (PI3K) play an essential role in signal transduction through tyrosine kinase- and heterotrimeric G-protein-linked receptors. In the family of class I kinases, PI3Kδ activity is restricted to leukocytes and activated through a variety of receptors, such as cytokine, growth factor, and antigen receptors [108, 109]. PI3Kδ consists of a catalytic subunit (p110δ) and a regulatory subunit (p85α), which are encoded by the *PIK3CD* and *PIK3R1* genes, respectively [109]. PI3Kδ is responsible for the generation of the second messenger molecule phosphatidylinositol 3,4,5-trisphosphate (PIP_3_) by phosphorylating its precursor phosphatidylinositol 4,5-bisphosphate (PIP_2_). Downstream PIP_3_ signaling is mediated by intracellular enzymes, such as the serine/threonine kinase AKT, which phosphorylates the FOXO transcription factors inactivating them, as well as regulators of the mTOR complex 1 (mTORC1), which is in turn activated [109].

**Fig. 5:**
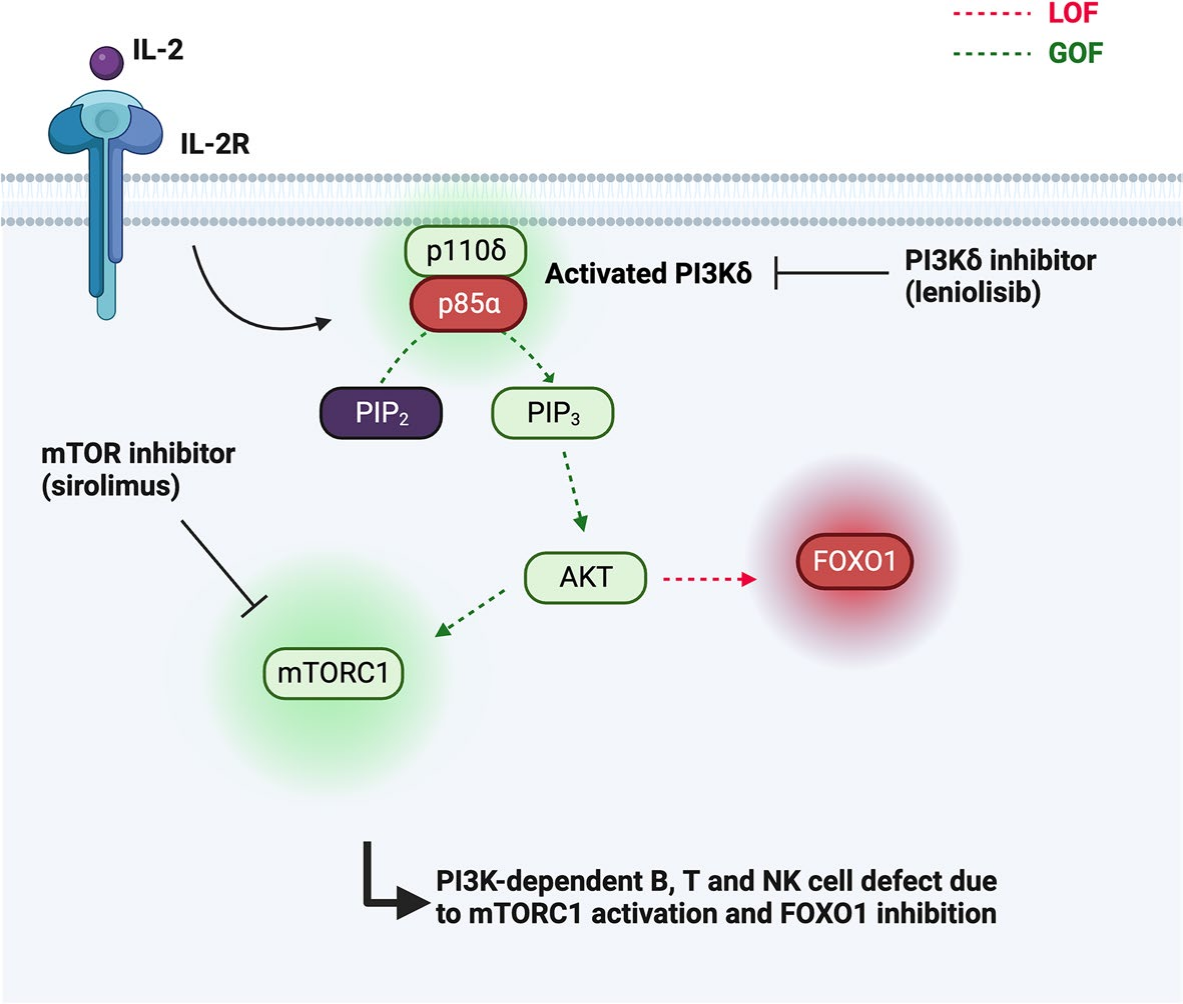
Illustration of the PI3Kδ pathway. PI3Kδ is activated through a variety of receptors (shown here is activation via IL-2 and its associated receptor). PI3Kδ typically consists of a catalytic subunit (p110δ) and a regulatory subunit (p85α) and leads to the generation of phosphatidylinositol 3,4,5-trisphosphate (PIP3) by phosphorylating its precursor phosphatidylinositol 4,5-bisphosphate (PIP2), both located in the cell membrane. Downstream PIP3 signaling is mediated by intracellular enzymes, such as the serine/threonine kinase AKT, which phosphorylates the FOXO transcription factors inactivating them, as well as regulators of the mTOR complex 1 (mTORC1), which is in turn activated. Increased activity of the PI3Kδ pathway leads to APDS. Patients with APDS are responsive to mTOR inhibition. More targeted approaches include selective PI3Kδ inhibitors, such as leniolisib or idealisib

Increased activity of the PI3Kδ pathway leads to an autosomal dominant primary immunodeficiency called APDS (Fig. 5). Two entities have been described: APDS1 due to heterozygous GOF mutations in the *PIK3CD* gene [8, 9] and APDS2 resulting from heterozygous LOF mutations in the *PIK3R1* gene [10, 11], which are phenotypically similar due to an overall increase in PI3Kδ activity (“gain-of-PI3Kδ-activity” mutations). Compared to other ALPID conditions, APDS has higher clinical penetrance and lower genetic heterogeneity [110]. Thus, more than 90% of APDS1 patients carry the *PIK3CD* E1021K mutation. *PIK3R1* encodes the subunits p85α, p55α, and p50α as alternative splicing products; thus, loss-of-function mutations may cause different splice variants and consequently affect PI3Kδ signaling and disease phenotype [111].

A hallmark of APDS is recurrent respiratory tract infections as early as in the first year of life in almost all patients, as well as early-onset bronchiectasis (especially in APDS1) [110, 112–114]. Lymphadenopathy, hepatosplenomegaly, and recurrent/chronic herpesvirus infections, as well as a variety of autoimmune manifestations such as cytopenia, glomerulonephritis, primary sclerosing cholangitis, or inflammatory bowel disease can be observed [8, 10, 11, 112, 113, 115]. Some non-immunologic complications are developmental delay and growth impairment, particularly in APDS2 patients [112, 113]. The median age of onset is below 2 years of age [110].

Patients often have increased IgM and normal to decreased IgA and total IgG levels, which prior to the first description of APDS often led to the incorrect diagnosis of hyper IgM syndrome (HIGM) [116]. The observed hypogammaglobulinemia might be due to a PI3K-dependent B cell intrinsic defect (e.g., B cell survival [117]), extrinsic defect (e.g., increased AICD of T lymphocytes [8]), or a combination of both [11]. Moreover, increased PI3Kδ signaling has been shown to impair antibody maturation, since it actively suppresses class-switch recombination, e.g., through FOXO inactivation [118]. B cell counts in most patients are progressively decreasing with an expansion of transitional B cells and reduction of class-switched B cells [112, 119]. In terms of T cell phenotype, CD4 T cell counts are reduced, especially CD45RA + T cells [112]. mTORC1 signaling has been shown to differentially regulate the development of T effector and regulatory cells [120], with overactivation of the AKT/mTOR pathway leading to a burst in proliferation and accumulation of terminally differentiated and senescent effector T cells including expansion of CD57 + CD8 + T cells by various mechanisms, such as a metabolic bias towards glycolysis [121, 122]. Susceptibility to herpes infection, especially EBV [112, 123], was shown to develop due to reduced cytotoxicity of the exhausted/senescent CD8 + T and the abnormally differentiated NK cells [124, 125]. For functional testing, mutated p110δ or p85α can be expressed in a cell line and lipid kinase activity measured via a membrane capture assay [126]. A more common functional assay is the measurement of AKT and S6 phosphorylation, reflecting the activated PI3Kδ pathway [127].

Despite high phenotypic overlap, APDS1 patients more commonly present with bronchiectasis, splenomegaly, cytopenia, and skin disease, whereas APDS2 patients develop growth impairment and lymphoma more frequently [110]. Ten to 15% of patients developed malignant disease at a median age of 19 years, most commonly diffuse large B cell lymphoma (DLBCL) and classical Hodgkin lymphoma, with prior EBV infection posing a risk for malignant lymphoproliferation [110]. However, an even more important oncogenic driver is the increased PI3Kδ/AKT/mTOR signaling leading to B and T cell defects [128, 129]. In the largest cohort to date (the ESID APDS registry), death occurred at a median age of 18.5 years [110].

### Treating ALPID: basic concepts and targeted therapies

Most patients with ALPID benefit from conventional PID treatments. Since they often present with hypogammaglobulinemia and/or respond poorly to vaccines, many are treated with immunoglobulin replacement therapy to correct for the secondary antibody deficiency [130]. If recurrent bacterial infections are present, this conventional therapy is often supplemented with prophylactic antibiotics with the aim of reducing infection susceptibility and secondary complications such as chronic lung inflammation and bronchiectasis.

Next to infection susceptibility, many inborn errors of immunity present with immune dysregulation [131]. The basic pillars of the treatment of autoimmunity and inflammation are steroids and other non-selective immunosuppressants, such as mycophenolate mofetil (MMF), azathioprine, and cyclosporine. Monoclonal antibodies (e.g., anti-TNF, anti-IL-17) are used for the treatment of inflammatory bowel disease or inflammatory skin disease. The use of these substances usually follows the standard recommendations, but the increased infection susceptibility of ALPID patients has to be carefully considered.

In recent years, targeted therapies have become available for several autoimmune-lymphoproliferative diseases. Rapamycin (sirolimus) is an mTOR inhibitor that can be considered a targeted therapy for ALPS patients [132]. mTOR inhibition targets DNT hyperproliferation and inappropriate survival of autoreactive B cells [133]. After the initial successful treatment of ALPS with rapamycin [134, 135], further studies could show rapid improvement of non-malignant lymphoproliferation and autoimmune cytopenia, which was mirrored in a decrease in DNT cells and ALPS biomarkers [133, 136]. Rapamycin is increasingly used as a first-line treatment for ALPS and shows amazing efficacy in this disease. It is often used as an immunosuppressive agent in CTLA-4 haploinsufficiency and LRBA deficiency where it can improve both lymphoproliferation and autoimmunity, especially enteropathy [70, 137].

Since mTOR signaling is activated downstream of PI3Kδ, patients with APDS also benefited from the use of rapamycin, especially in controlling benign lymphoproliferation. However, non-lymphoproliferative complications (cytopenia, enteropathy) are less responsive to mTOR inhibition [112–114, 138].

The development of selective PI3Kδ inhibitors, such as leniolisib or idealisib, allows for a specific inhibition of the hyperactive PI3Kδ pathway in APDS patients. A clinical trial of leniolisib (NCT02435173) or seletalisib (European Clinical Trials Database 2015–002900-10), oral inhibitors of the p110δ subunit, showed a decrease in lymphoproliferation and some improvement in autoimmune complications (cytopenia) [127, 139, 140]. Treatment was well tolerated with very little side effects. Notably, the treatment also resulted in the normalization of several features of the abnormal immune phenotype, allowing to stop immunoglobulin substitution in some patients.

Targeted treatment is also available for patients with CTLA-4 haploinsufficiency or LRBA deficiency. The soluble CTLA-4 immunoglobulin fusion protein abatacept consisting of the Fc-region of human IgG1 linked to the extracellular domain of CTLA-4 can mimic CTLA-4 function. It has been shown to successfully control lymphoproliferation and many autoimmune manifestations, such as cytopenia and enteropathy [70, 137, 141]. In a cohort of 123 CTLA-4 haploinsufficient patients, abatacept led to an improvement of interstitial lung disease and enteropathy with a response rate above 70% [137]. However, steroids continue to play an essential role in disease management and abatacept often needs to be combined with other immunosuppressive agents, especially if symptomatic lymphoid infiltrates are present. Moreover, abatacept may lead to viral reactivation; therefore, EBV and CMV viremia should be routinely monitored [63]. Data on long-term disease control under abatacept are still lacking.

Although hyperactive STAT3 cannot be targeted directly, other molecules which are part of the STAT3 pathway can be inhibited. Disruption of the IL-6/IL-6R interaction upstream of STAT3 via the anti-IL-6R monoclonal antibody tocilizumab was shown to partially improve autoimmunity [82, 85] by increasing FoxP3 + Treg cells [142]. However, small molecule inhibitors of JAK activation (jakinibs such as ruxolitinib) lead to an even more impressive control of autoimmunity and immune dysregulation, especially if initiated early [85, 143]. Whether Jakinib therapy represents a life-long treatment concept for STAT3 GOF disease remains to be evaluated.

For ALPID patients, hematopoietic stem cell transplantation (HSCT) is usually restricted to patients with early onset of disease, severe disease manifestations of critical organs (lung, brain), and insufficient response to treatment, including targeted therapies. The hyperactive signaling pathways, resulting in autoimmunity and hyperinflammation, frequently combined with persistent viral infections, poses a great challenge for successful stem cell transplantation [144]. Especially when conventional treatments fail, HSCT is the only long-term curative therapy. There have been multiple retrospective studies showing an improvement in disease manifestations, e.g., in APDS [145, 146], CTLA-4 haploinsufficient [137], LRBA deficient [70], and STAT3 GOF [84, 143] patients. In an initial cohort of 23 STAT3 GOF patients, overall survival was 62% [84]. Curative HSCT in patients with LRBA deficiency showed an overall survival rate of 70.8%, and all deaths could be attributed to a short-term transplant-related mortality [70]. Moreover, more than 2/3 (70.6%) of those patients required no further immunosuppressive therapy, which was in stark contrast to non-transplanted LRBA-deficient patients [70]. Similar results were reported regarding CTLA-4 haploinsufficiency: 13 out of 18 transplanted patients (72.2%) remained disease-free with only one requiring IRT [137]. Overall survival in APDS1 and APDS2 was 86% in a cohort of 57 patients, with poor graft performance being the most common complication [146]. Based on these retrospective data, primary HSCT is a potentially curative treatment option in patients presenting with an ALPID phenotype. However, additional data are needed to provide criteria for the selection of suitable patients and the right time point based on risk factor stratification.

### Outlook: variable penetrance and somatic mutations

These examples illustrate the clinical and pathophysiological overlaps and differences of selected ALPID diseases. Even within a defined genetic condition, clinical heterogeneity makes diagnosis and management a challenge (Table 1). Moreover, many patients with similar clinical and immunological presentation remain without a genetic diagnosis, despite significant advances in next-generation sequencing (NGS), particularly whole exome sequencing (WES) [147]. Several recent examples have shown that somatic mutations providing a proliferative advantage to lymphocytes can lead to complex ALPID phenotypes. Genetic analysis must pay particular attention to such non-mendelian constellations, which require deeper sequencing efforts than currently offered by exome or genome analysis.

**Table 1:** Comparison of the ALPID syndromes

Disease	ALPS	CTLA-4 haploinsufficiency and LRBA deficiency	STAT3 GOF	NF-kB1 haploinsufficiency	APDS
Genes	*FAS*, *FASLG*, *FADD*	*CTLA4*, *LRBA*	*STAT3*	*NFKB1*	*PIK3CD, PIK3R1*
Pattern of heredity	Variable inheritance, somatic variants (LOF)	*CTLA4*: autosomal-dominant, *LRBA*: autosomal-recessive (LOF)	Autosomal-dominant, somatic variants (GOF)	Autosomal-dominant (LOF)	Autosomal dominant, gain-of-PI3Kδ-activity
Genetic heterogeneity	High	High	High	High	Low (E1021K hotspot in *PIK3CD*—90% of cases)
Penetrance	Incomplete	CTLA-4: incomplete, LRBA: complete	Incomplete	Incomplete	Almost complete
Mechanism	Defective extrinsic apoptotic pathway	Lack of CTLA-4 and decreased Treg function	Increased STAT3-dependent transcription, decreased function of other STATs	Reduced levels of p105/p50 subunit	Increased activity of PI3Kδ with mTORC1 activation and FOXO1 inhibition
Age of onset	Variable, majority in childhood	CTLA-4: median 11 years, LRBA: infancy	Median 2,3 years	Highly variable, median 12 years	Median 1 year
Most common first symptom(s)	Lymphoproliferation	Immune dysregulation	Immune dysregulation	Infections	Infections
Autoimmunity	Cytopenia	Cytopenia, enteropathy, encephalitis	Cytopenia, enteropathy, diabetes, growth failure	Cytopenia, inflammatory disease	Cytopenia, enteropathy
Lymphoproliferation	Splenomegaly, lymphadenopathy + +	Splenomegaly, lymphadenopathy	Splenomegaly, lymphadenopathy	Splenomegaly, lymphadenopathy	Splenomegaly, lymphadenopathy + + + , mucosal lymphoid hyperplasia
Immunoglobulins	Hyper-IgG, 5–10% hypogammaglobulinemia	Frequent hypogammaglobulinemia	Sometimes hypogammaglobulinemia	Frequent hypogammaglobulinemia	Increased IgM, sometimes hypogammaglobulinemia
Recurrent infections	Not prominent	Yes, interstitial lung disease	Yes, interstitial lung disease	Yes	Yes, early-onset bronchiectasis
Immunological findings	Expansion of DNT, sometimes low class-switched B cells	Increased Tfh, increased CD21low, decreased class-switched B cells	Increased CD21low B cells, reduced Treg	Increased CD21low B cells, decreased T cell function	Increased transitional, reduced class-switched B cells, increased senescent T cells
Disease-specific assay	ALPS biomarkers	Trans-endocytosis assay	STAT3 reporter assay	NF-kB1 reporter assay	S6 phosphorylation
Targeted therapy	mTOR inhibitor	CTLA-4 fusion protein	JAK inhibitor, anti-IL-6R monoclonal antibody	-	PI3Kδ inhibitor

Another fascinating research topic is the variable clinical penetrance of these mostly autosomal dominant conditions. While some cases may be explained by second-hit somatic mutations leading to clinical manifestation of the disease, other factors that need to be considered are other genetic or epigenetic factors or environmental factors such as microbiota or metabolic cues. A better understanding of penetrance factors may result in prophylactic measures and will allow better discussion of prognosis for affected patients.

All figures were created via BioRender.

## Data Availability

Not applicable.

## Abbreviations

AICD: Activation-induced cytidine deaminase
AICD: Activation-induced cell death
ALPID: Autoimmune lymphoproliferative immunodeficiencies
ALPS: Autoimmune lymphoproliferative syndrome
APDS: Activated PI3Kδ syndrome
APC: Antigen-presenting cells
BCR: B cell receptor
CD: Cluster of differentiation
CTLA-4: Cytotoxic T lymphocyte-associated antigen-4
CVID: Common variable immunodeficiency
DISC: Death-induced signaling complex
DNT: Double-negative T cells
FADD: Fas-associated death domain
FoxP3: Forkhead box P3
GOF: Gain of function
HIES: Hyper-immunoglobulin E syndrome
HIGM: Hyper IgM syndrome
HSCT: Hematopoietic stem cell transplantation
IFN: Interferon
Ig: Immunoglobulin
IL: Interleukin
IKK: IκB kinase
JAK: Janus kinase
LOF: Loss of function
LOH: Loss of heterozygosity
LRBA: Lipopolysaccharide-responsive vesicle trafficking, beach- and anchor-containing
MHC: Major histocompatibility complex
mTOR: Mammalian target of rapamycin
mTORC1: MTOR complex 1
NF-κB: Nuclear factor kB
PI3K: Phosphoinositide 3-kinases
PIAS3: Protein inhibitor of activated STAT3
PIP2: Phosphatidylinositol 4,5-bisphosphate
PIP3: Phosphatidylinositol 3,4,5-trisphosphate
PRR: Pattern-recognition receptors
sFasL: Soluble Fas ligand
SOCS3: Suppressor of cytokine signaling 3
STAT: Signal transducer and activator of transcription
TCR: T cell receptor
Tfh: T follicular helper cells
TNF-R: Tumor necrosis factor-receptor
Treg: Regulatory T cells
WES: Whole exome sequencing
